# Enteric Fever: Diagnostic Challenges and the Importance of Early Intervention

**DOI:** 10.7759/cureus.41831

**Published:** 2023-07-13

**Authors:** Tias Saha, Abimbola E Arisoyin, Bhaswanth Bollu, Tejaswini Ashok, Athira Babu, Ali Issani, Sharan Jhaveri, Chaithanya Avanthika

**Affiliations:** 1 Internal Medicine, Samorita General Hospital, Faridpur, BGD; 2 Internal Medicine, Diabetic Association Medical College, Faridpur, BGD; 3 Internal Medicine, University of Lagos, Lagos, NGA; 4 Emergency Medicine, All India Institute of Medical Sciences, New Delhi, IND; 5 Internal Medicine, Jagadguru Sri Shivarathreeshwara Medical College, Mysore, IND; 6 Pediatrics, Saudi German Hospital, Dubai, ARE; 7 Emergency Medicine, Aga Khan University, Karachi, PAK; 8 Internal Medicine, Nathiba Hargovandas Lakhmichand Municipal Medical College, Ahmedabad, IND; 9 Pediatrics, Icahn School of Medicine at Mount Sinai, Elmhurst Hospital Center, New York, USA; 10 Medicine and Surgery, Karnataka Institute of Medical Sciences, Hubli, IND

**Keywords:** surveillance, typhoid vaccines, serological tests, chronic carrier, multidrug resistant salmonella

## Abstract

Enteric fever is a systemic infection caused by highly virulent *Salmonella enterica *serovars: Typhi and Paratyphi. Diagnosis of enteric fever is challenging due to a wide variety of clinical features which overlap with other febrile illnesses.

The current diagnostic methods are limited because of the suboptimal sensitivity of conventional tests like blood culture in detecting organisms and the invasive nature of bone marrow culture. It emphasizes the need to develop improved and more reliable diagnostic modalities. The rising rates of multidrug-resistant *Salmonella* strains call for an accurate understanding of the current management of the disease. Proper public health measures and large-scale immunization programs will help reduce the burden of the disease. A comprehensive surveillance system can help detect the chronic carrier state and is crucial in understanding antibiotic susceptibility patterns.

We conducted an all-language literature search on Medline, Cochrane, Embase, and Google Scholar till May 2022. The following search words and medical subject headings (MeSH) were used: "enteric fever," "*Salmonella* Typhi," "multidrug-resistant *Salmonella*," chronic carrier state," "*Salmonella* detection, "and "typhoid vaccine." We reviewed the literature on clinical features, pathophysiology, new diagnostic tests, and interventions to prevent the disease.

This article explores enteric fever and its various clinical features and addresses the emerging threat of multidrug resistance. It focuses on novel methods for diagnosis and prevention strategies, including vaccines and the use of surveillance systems employed across different parts of the world.

## Introduction and background

Enteric fever is a systemic infection caused by *Salmonella enterica* (*S. enterica*) serotypes, Typhi (most common) and Paratyphi A, B, and C. It attributes to significant morbidity and mortality in resource-limited countries like Asia, Africa, and South America with a limited potable water supply and improper sanitation [1]. According to WHO, annually 21 million enteric fever cases and up to 161,000 deaths are reported worldwide [1]. However, a recent study about the global burden of typhoid and paratyphoid fever in 2017 reported a 41% decline in deaths from enteric fever compared to 1990 [2].

Transmission of *Salmonella* Typhi (*S.* Typhi) and *Salmonella* Paratyphi (*S.* Typhi) occurs fecal-orally from contaminated food or water. There is a short-cycle transmission in which the bacteria contaminate the environment from acute or chronic carriers due to inadequate sanitation, while pollution of big water bodies by sewage is a long-cycle transmission [3].

Signs and symptoms of enteric fever are wide-ranging and mimic other systemic illnesses. It includes fever, chills, headaches, anorexia, abdominal discomfort, relative bradycardia, vomiting, diarrhea, constipation, hepatomegaly, splenomegaly, leukopenia, and thrombocytopenia. The clinical features and complications can also vary according to age, making a clinical diagnosis of enteric fever difficult [4,5]. Common complications include anemia, gastrointestinal bleeding, intestinal perforation, bone marrow hypoplasia, encephalopathy, disseminated intravascular coagulation, and shock. The reports showed that the 10-30% case fatality rate drops to 1-4% with treatment [4]. A study reviewing 83 reports of enteric fever cases from South Asian countries showed that preschool children (6%) had the highest case fatality rates compared to other age groups [5]. Hence, it is imperative to diagnose enteric fever early to prevent mortality. Furthermore, 2-5% of patients, even after recovery, become asymptomatic carriers and continue to shed the bacteria [6,7].

The current case definition of enteric fever is fever ≥38 °C for at least three days with a positive culture of bacteria from blood/bone marrow. However, several factors are limiting the definitive diagnosis: the volume of blood required (usually more than 7 mL), depending on the age of the subjects due to low levels of bacteremia (we also need to transport blood at ambient temperature (15-40˚c)); low sensitivity ~50% for blood culture and ~80% for bone marrow culture, even though the specificity reaches 100% for both; and lack of resources, including trained personnel and laboratory equipment.

According to WHO, the gold-standard diagnosis of typhoid fever should approach 100%, each for sensitivity, specificity, and positive and negative predictive values [1,8].

The recommended antimicrobial treatment for enteric fever includes chloramphenicol, ampicillin, trimethoprim-sulfamethoxazole, fluoroquinolones, third-generation cephalosporins, and azithromycin. The recent emergence of multidrug-resistant (MDR) *Salmonella* has limited the use of many of these drugs. Many mechanisms, such as acquiring chromosomal mutations by a transposon and plasmid exchange cause the resistance, and haplotype-58(H58) is the most dominant strain [1,8].

There are two vaccines licensed and accessible for enteric fever which is summarized in Table 1. The choice of a vaccine depends on the target age group, vaccine efficacy, patient compliance, and doctor's preference.

**Table 1:** Types of typhoid vaccines

Vaccine	Route	Number of doses	Age for administration	Type of vaccine
Vi capsular polysaccharide vaccine (Vi-Ps)	Injectable	Single dose	Above two years	Subunit antigen
Ty21a strain vaccine	Oral	Three doses	Above five years	Live attenuated

A study in Chile reported 67% of accumulative efficacy after three doses of oral vaccine (one capsule every other day) at three years and 62% at seven years of follow-up [9,10].

In conjunction with routine immunizations, interventions like improving water quality and sanitation and supervision of antimicrobial resistance followed by reporting the antimicrobial susceptibility data can help in mitigating the mortality and morbidity burden of enteric fever worldwide [11].

## Review

Pathophysiology

Enteric fever, one of the foremost bacterial infections worldwide, is mainly caused by *S. enterica* serovar Typhi [12]. *S. enterica* serovars Typhi, Paratyphi A, Paratyphi B, and Paratyphi C are called typhoidal *Salmonella* serovars because Paratyphi strains cause similar clinical symptoms [13,14]. *S. *Typhi and Paratyphi are pathogens restricted to humans and not common in other *Salmonella* serovars. *S. *Typhi exists as a Gram-negative encapsulated, flagellated, facultative anaerobic bacilli. It possesses three major antigens: H or flagellar antigen, O or somatic antigen, and Vi antigen [15].

The infection transmits through poor hygiene and the fecal pollution of food and water [16]. The incubation period is mostly 7-14 days. After establishing an initial, clinically undetectable infection and transient primary bacteremia, the organism disseminates systemically to the liver, spleen, bone marrow, Peyer’s patches of the terminal ileum, and gallbladder [17,18]. Large numbers of bacteria then spill into the bloodstream, initiate secondary bacteremia, and manifest definite clinical symptoms.

**Figure 1:**
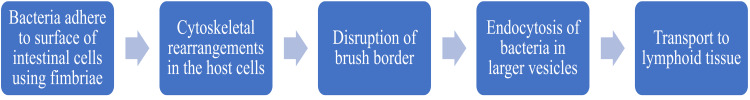
Pathogenesis of typhoid

Typhoid induces mucosal, humoral, and cellular immune responses, but these do not grant complete protection in reinfection and relapse [19]. Specialized epithelial M cells in Peyer’s patches are the potential site of invasion of *Salmonella* by a pathologically distinct process, unlike the conventional receptor-mediated endocytosis [20]. The pathogenesis of typhoid is illustrated in Figure 1 [21].

The pathological hallmark of enteric fever is mononuclear cell infiltration and hypertrophy of the reticuloendothelial system, including the intestinal Peyer's patches and mesenteric lymph nodes [22]. Recent data showed another alternative method used by *Salmonella* to disseminate from the gastrointestinal tract by passively traversing the epithelial barrier using CD18-positive phagocytes [23].

Salmonella Pathogenicity Islands

Different pathogenic *Salmonella* strains have evolved by obtaining a group of virulence (Vi) genes in a contained area of the chromosome called *Salmonella* pathogenicity islands (SPIs) [24]. Diverse SPIs of variable sizes showed that they encrypt many Vi factors that help the organism survive, adhere, invade, and produce toxins.

*S. *Typhi encodes four relatively distinct SPIs: SPI-7, 8, 15, and 18, and these SPIs have a significant contribution to Vi. SPI-7 encodes Vi capsule, type IV B pilus, and via B locus [25]. The Vi capsule holds vital immunomodulatory functions such as dampening immune response, escaping peroxide-mediated killing, and preventing complement activation [26,27]. SPI-11- encodes typhoid toxin.

Type III Secretion System

Several pathogenicity islands, including SPI-1 and SPI-2, encrypt specialized tools to inject Vi proteins into host cells, termed type III secretion system (TTSS), which are distinct Vi phenotypes of each pathogen [28]. These injected bacterial components change fundamental host-cell functions like signal transduction, membrane trafficking, and cytoskeletal arrangement. Extracellularly, the SPI-1 TTSS helps to attack non-phagocytic cells and induce intestinal inflammatory responses [29,30]. On the contrary, the SPI-2 TTSS comes into action after internalization and helps to promote the development of the *Salmonella*-containing vacuole (SCV) and intracellular replication [31]. Amusingly, several *Salmonella* TTSSs genes are present within temperate bacteriophages, which indicates that horizontal gene transfer allows fine-tuning of Vi phenotypes by mixing different TTSS effector proteins [32,33].

Pathogen-Associated Molecular Patterns

*Salmonella* strains express distinct structures on their surface, such as Toll-like receptors (TLR) and NOD-like receptors. They are pathogen-associated molecular patterns (PAMPs) [34]. Naturally, host innate immunity can identify PAMPs; however, *S. *Typhi Vi capsular polysaccharide and the typhoid toxin do not trigger a pro-inflammatory response by hindering the PAMPs and, thereby, allowing them to withstand intracellular development in the reticuloendothelial system [35]. After invading the intestinal cells, macrophages in the lamina propria overtake the bacteria, and regardless of bactericidal activities in host cells, *S. *Typhi can persist and replicate in macrophages using SPI-2. *S. *Typhi also triggers via B locus expression and silences flagella expression to prevent TLR recognition and successive IL-8 production [36,37]. Thus, one of the mechanisms by which *S. *Typhi escapes the adaptive immune system is lowering TLR signaling, which halts the induction of the IL-12/IFN-γ axis of lymphocytes.

Typhoid Toxin

Another Vi factor linked with typhoidal *Salmonella* is an atypical AB toxin, secreted within vesicles originating from SCV and released into the extracellular space, where it binds to Neu5Ac-terminated receptors on target cells inducing G2/M cell cycle arrest and cell death [38,39]. Following export, typhoid toxin infects various cells through autocrine and paracrine pathways [39]. *S. *Paratyphi A lacks Vi capsule, but they can intricate long O-chains, which helps to avoid antibody-mediated complement activation [40].

The non-typhoidal *Salmonella* serovars (NTS) (e.g., *S. *Enteritidis and *S. *Typhimurium), which primarily cause gastroenteritis also infect the same route. However, their clinical and pathological courses are quite different. While NTS gastroenteritis has a massive neutrophil influx which keeps the infection localized and restricts it to a short course of symptoms, enteric fever does have a long course of illness with delayed onset. Additionally, the invasion of macrophage-like cells with *S. *Typhi results in noticeably low production of the neutrophil chemoattractant interleukin (IL)-8 compared to invasion with *S. *Typhimurium [41]. These findings suggest that *S. *Typhi holds unique Vi traits that allow it to down-regulate pattern recognition receptors (PRRs) mediated host response in the intestinal mucosa [42].

Threatening the emergence of multidrug resistance strains

A major revolution in the treatment of enteric fever was the introduction of chloramphenicol in 1948. Ampicillin and trimethoprim/sulfamethoxazole were used as alternatives, whereas chloramphenicol use was not applicable [43]. However, chloramphenicol was used widely and often erratically due to its orally available form, broad-spectrum range, and cheap rate resulting in the blooming of resistance.

The efficacy of chloramphenicol remained pleasing until 1989; however, there was a quick emergence of MDR *S. *Typhi (resistant to three first-line antibiotics recommended by the WHO: chloramphenicol, ampicillin, and trimethoprim-sulfamethoxazole) in several parts of India [44]. A study conducted across typhoid-endemic Asian countries reported that the prevalence of MDR *S. *Typhi strains climbed from 7% to 65% [45]. Studies show IncHI1 plasmids embrace various genes on composite transposons in the MDR strains approaching high transmissibility [46,47]. Possession of the R plasmid encoding acetyltransferase enzyme is the reason for chloramphenicol inactivation, which results in resistance.

The genes responsible for ampicillin and trimethoprim-sulfamethoxazole resistance are TEM-1 β-lactamase and dihydrofolate reductase type VII. MDR enteric fever is more dangerous because its complication and mortality rate is higher than sensitive strains. High mortality rates may be related to the increased Vi of multidrug-resistant *Salmonella* and a higher number of circulating bacteria [48].

Resistance to older antibiotics has forced researchers to develop the next best option to combat salmonellosis, including fluoroquinolones (mainly ciprofloxacin) and extended-spectrum cephalosporins [49,50]. Fluoroquinolones aim for DNA gyrase and topoisomerase IV, the bacterial enzymes playing a major role in their DNA transcription. For moxifloxacin and gatifloxacin resistance, the primary target of bacteria is the gyrA gene mutation (which encodes DNA gyrase). For levofloxacin and ciprofloxacin, it is the parC gene (encodes topoisomerase IV) [51,52]. Whole-genome sequence analysis of diverse *S.* Typhi isolates revealed the spread of a single *S.* Typhi haplotype, termed H58 (or genotype 4.3.1.), which is highly responsible for multidrug resistance and reduced fluoroquinolone susceptibility [53,54]. A steady increase noticed in the minimum inhibitory concentration (MIC) of fluoroquinolones was due to the rapid emergence of the H58 S. Typhi genotype in South Asia. Currently, it is no longer considered a first-line typhoid treatment [55-57].

In reply to the rise of fluoroquinolone resistance, third-generation cephalosporins have been a rescuer in treating enteric fever [58]. Still now, ceftriaxone and cefotaxime have remained trustworthy antimicrobial resources specifically for inpatient cases [59]. While the term MDR described resistance to three first-line antibiotics, extensive drug resistance (XDR) exhibited resistance to fluoroquinolones and third-generation cephalosporins in addition to first-line antibiotics [44]. *S.* Typhi holds the capability to alter from MDR to XDR by cherishing an IncY plasmid, which also confers resistance to fluoroquinolones. In addition, horizontal acquisition of this plasmid produces extended-spectrum beta-lactamases (ESBLs) through the blaCTX-M-15 gene can result in cephalosporin resistance [60]. Indiscriminate and offhand use of these cephalosporins led to the advancement of varied ESBL types such as PER, SHV, and TEM enzymes [61,62]. Reports of a high sporadic ceftriaxone resistance validate this statement [25]. The advent of ESBLs in MDR strains indicates urgent reconsideration of the use of cephalosporins, especially in developing countries [63].

If the MDR strains have intermediate susceptibility or resistance to ciprofloxacin, oral Azithromycin, a broad-spectrum azalide, is an effective alternative. It is very effective in removing intracellular *Salmonella*. Other beneficiary properties include low relapse rate, rapid defervescence, convalescent fecal carriage, favorable outpatient compliance, and pediatric suitability [64,65]. Although currently rare, *S.* Typhi strains with high azithromycin MICs and treatment failure have been stated [66]. Azithromycin resistance is due to the msrA, msrD, and ereA genes [53]. Nevertheless, if we encounter XDR strains during clinical practice, the treatment options become extremely limited to expensive drugs such as tigecycline or carbapenems (e.g., meropenem, imipenem, and ertapenem). However, minimal studies are currently present to show their efficacy in the treatment of enteric fever. This necessitates systematic, large-scale studies to assess the relative advantages of these drugs in treating enteric fever [67].

Antibiotic resistance has emerged as a massive challenge in the management of enteric fever, and threats to the growth of *S.* Typhi resistance consist of irrational prescription of antimicrobial combinations, easy accessibility of drugs at the pharmacy, unrestrained use of antibiotics in agriculture, and failure to trace chronic carriers [14,68].

The ideal management of enteric fever emphasizes the local pattern of antimicrobial resistance and antimicrobial susceptibility testing of the isolates [46]. Some forthcoming approaches to alleviate drug resistance in developing countries include judicious antibiotic prescribing practices by healthcare personnel and vaccination of at-risk people in endemic zones [48]. Interestingly, the re-emergence of chloramphenicol and co-trimoxazole vulnerable strains has shown the path for reintroducing the first-line antibiotics, but there are questionable concerns regarding their relapse [69,70]. Combination therapy might improve antibiotic-resistant states by synergistic action, but we have limited proof to validate this approach [71]. Moreover, as XDR strains flare up, novel therapeutic approaches like bacteriophage therapy will be needed to resist the infection [72,73].

Clinical manifestations, complications, and hostile chronic carrier state

Enteric fever is a common bacterial infection with nonspecific findings [1]. High-grade continuous fever (step ladder pattern) is the most essential and common presenting feature in the initial stage. The incubation period is 7-14 days as mentioned above. Patients may have nonspecific findings such as chills, abdominal pain, headache, constipation or diarrhea, weakness, dizziness, nausea, and cough. The severity of symptoms can fluctuate from being able to maintain normal activities of daily living to patients requiring inpatient care [74].

The patient may have diffuse abdominal tenderness, hepatosplenomegaly, and lymphadenopathy on physical examination. Rose spots, which appear during the second week of illness, are fine diffuse maculopapular rashes seen on the trunk and spreading to arms and legs. They are pathognomonic findings of enteric fever and are present in 3% to 40% of the patient. Some patients may also have relative bradycardia [75].

Feces represent the primary portal of exit of *S.* Typhi, although shedding in urine has also been documented [76]. Surveillance of patients with acute typhoid fever has demonstrated that up to 10% of the untreated patients excrete *S.* Typhi in their stools for up to three weeks following infection and are called chronic carriers. Carriers of *S.* Typhi can excrete between 10⁶ and 1010 organisms per gram of feces, a bacterial load that is large enough to promote fecal-oral transmission of the organism. People who work as food handlers comprise most of them [75].

Untreated acute enteric fever may result in complications (21-34%) irrespective of age. Patients may present with gastrointestinal tract complications, hematological, cardiovascular, pulmonary, and other organ systems.

Table 2 lists all the untreated or progressive infection complications according to the type of organ system involved [77].

**Table 2:** Complications of enteric fever ADH: antidiuretic hormone

Digestive system	Hepatitis, liver abscess, cholecystitis (predominantly acalculous type), paralytic ileus, ileal ulcer and gastrointestinal bleeding, intestinal perforation, pancreatitis
Blood and lymphatic system	Anemia, leukopenia, thrombocytopenia, bone marrow suppression (resulting in pancytopenia), disseminated intravascular coagulation, hemophagocytosis, splenic infarction, and abscess
Cardiovascular system	Myocarditis, sick sinus syndrome, and heart block
Central nervous system	Psychosis, and encephalopathy
Respiratory system	Acute respiratory distress syndrome, pneumonia, pleural effusion, bronchitis, and lung abscess
Musculoskeletal system	Osteomyelitis (in children with sickle cell disease) and arthritis/arthralgia
Endocrine	Syndrome of inappropriate ADH secretion
Miscellaneous	Soft tissue abscess and mycotic aneurysms

Enteric fever leads to hypertrophy of the reticuloendothelial system, but necrosis and ulceration are mostly limited to Peyer’s patches distribution. The lethal complications of typhoid fever are intestinal hemorrhage and perforation. Perforation is typically located in the region of the terminal ileum and occurs secondary to necrosis of Peyer’s patches. The typhoid intestinal perforation reported incidence is between 0.8% and 18% [78].

Diagnostic challenges, available options, and novel approaches

Low socioeconomic countries account for the highest burden of enteric fever, with more than 21 million episodes per year. However, this can be misleading due to the lack of accurate tools for diagnosis [79,80]. They prefer a clinical diagnosis that is often erroneous over diagnostic tests in this context. Nevertheless, even making a clinical diagnosis is a challenge because clinical manifestations such as elevated temperature and white blood cells, low platelets, fatigue, and anorexia are not exclusive to enteric fever [81,82]. Identical symptoms can occur in diseases like dengue virus, influenza virus, tuberculosis, malaria, typhus, or even leishmaniasis, which are endemic in many developing countries [83].

Conventional Salmonella Detection

The gold-standard diagnostic method for enteric fever is the blood or bone marrow culture. Fever more than 38°C for at least 72 hours in addition to positive blood or bone marrow culture is the definitive diagnosis of enteric fever [50]. Both blood and bone marrow culture have a 100% specificity and allow profiling of the antibiotic resistance of the organism [50]. However, it requires technical skills, expertise, and facility. It is not ideal in endemic areas where results are required rapidly. The sensitivity of blood culture is about 50%, while that of bone marrow is about 80% [50]. The wide-scale application of the bone marrow culture is not feasible because of its invasive nature.

Serological Detection

The serology tests for enteric fever will only suggest the presence of the infection. It is easy, provides results rapidly, and we use it mainly in endemic areas. A serological test for enteric fever identifies the presence of antigens, mainly the Vi, lipopolysaccharide O, and flagellar H antigens.

These antigens are also present in other *Salmonella* serovars; therefore, specificity is not 100%. The Vi antigen is positive in *S.* Typhi and *S.* Paratyphi C and negative in *S.* Paratyphi A and B [84]. It is helpful as a screening test for chronic carriers [85].

A constraint in the Vi antigen serology-based test is the recent identification of some *S.* Typhi that are Vi negative and certain conditions that must be present before the Vi-positive *S.* Typhi can express the antigen [86,87].

The a, b, c, and d are flagellar H antigens present in *S.* Paratyphi A, *S.* Paratyphi B, *S.* Paratyphi C, and *S.* Typhi, respectively. Other flagellar H types present on *S.* Typhi are the j and z66 [1,88].

Widal Test

Agglutination reaction between the flagellar H antigen, lipopolysaccharide O antigen, and antibodies specific to them in the serum is the basis of the widely used Widal test [89,90]. Ideally, the Widal test is positive when done twice with a difference in antibodies level of about fourfold. However, it is generally done once due to low resources in developing countries where enteric fever is endemic [91-93].

Rapid Diagnostic Tests

Newer rapid diagnostic methods that are also cheap and show promising indicators include the Typhidot and Tubex TF, produced in Malaysia and Sweden. SD Bioline, Mega *Salmonella*, and Typhidot M are the new versions, but there is little information regarding their reliability [94-97].

Typhidot uses the enzyme-linked immunosorbent assay (ELISA) technique, which allows IgG and IgM against the 50kd outer membrane protein of the *S.* Typhi present in the serum of the patient. On the other hand, Tubex TF utilizes the reaction between the monoclonal antibodies, which are bound to the 09 lipopolysaccharides of the *S.* Typhi, and IgM antibodies present in the patient’s serum.

Biomarkers

A promising method of diagnosis involves investigating a series of new biomarkers by seeking the different unique biomarkers found in the plasma of patients with acute enteric fever. These methods of diagnosis show increased sensitivity and specificity [98-101]. They can distinguish acute infection from previous infection and vaccination. A significant challenge is the lack of a single perfect method; using a composite reference standard that combines multiple diagnostic methods can be helpful to mitigate this [102]. Intensified efforts to identify early infection biomarkers and differentiate chronic disease from ongoing active infection will be helpful.

Spectrometry

Studies show the different changes in specific metabolites in the plasma of a patient infected with enteric fever and a healthy person. Many gas chromatography-mass spectrometry studies showed clear peaks in 695 metabolites compared to their controls [103], and among them, the top six metabolites (ethanolamine, gluconic acid, monosaccharide, phenylalanine, pipecolic acid, and saccharide) were studied. These showed a clear difference in the plasma of typhoid fever when compared to healthy people and paratyphoid fever infection. An upregulation of hepcidin causing a fast reduction in serum iron levels and disruption of tryptophan breakdown is also closely related to *S.* Typhi infection [104,105].

Immunological Assay

Identifying different protein markers is also one of the newer approaches to diagnosing enteric fever. An ELISA and immunoblot-based test, the typhoid paratyphoid test (TPTest), compares IgA levels against *S.* Typhi and *S.* Paratyphi membrane components [69,99,106]. In patients with suspected typhoid, other illnesses, and healthy controls, the TPTest is applicable.

Furthermore, immunodominant and immunogenic antigens related to enteric fever have been studied using protein microarrays and immunoaffinity proteomics technology [107-110]. Mass spectrometry-based proteomics usually follows these processes, showing the bacteria bound to the proteins. Other studies have analyzed antigens using liquid chromatography-mass spectrometry with confirmation by western blotting. OmpA, OmpC, PagC, and HlyE are a few of the identified immunogenic antigens of the bacteria.

Molecular Assay-Polymerase Chain Reaction

A newer promising method of diagnosing enteric fever involves identifying and amplifying nucleic acid biomarkers using polymerase chain reaction (PCR), which involves a signature comprised of five host genes, namely STAT1, SLAMF8, PSME2, WARS, and ALDH1A1. This test had a 97% sensitivity and 88% specificity for enteric fever [111]. Microarray hybridization, RNA sequencing, and identifying mRNA using magneto-DNA probes are other various ways of detecting nucleic acid biomarkers.

Various DNA detection using PCR assays majorly focuses on the flagellin gene while using different bases to enhance the sensitivity. The flagellin gene is flic-a for *S.* Paratyphi A and flic-d for *S.* Typhi [112-116]. It appears to be promising not only because of the increased sensitivity and specificity but also because of the ability of PCR to amplify the DNA from dead bacteria.

Early interventions, preventive strategies, and the value of vaccination

Enteric fever requires punctual and efficient resolution to prevent complications, relapse, chronic carrier state, and death. The rising rates of antibiotic resistance worldwide have caused stimulated interest in a comprehensive strategy for the control of the disease. Many efforts are made globally to monitor public health and introduce large-scale immunization programs to mitigate the burden of the disease [117].

Public Health Measures

According to WHO reports in 2017, 2 billion people in Sub-Saharan Africa and Asia still lack access to basic sanitation facilities, and 18% of the worldwide population (1.4 billion people) has no access to handwashing facilities [118]. Typhoid fever control requires a robust surveillance system to estimate the disease load and preventive measures such as water, sanitation, hygiene, and personal hygiene awareness [74]. Providing safe drinking water, adequate sanitation, and safe food handling procedures are critical for preventing enteric fever [76]. To prevent the spread, the importance of handwashing before preparing food, eating, or feeding children is well ascertained [76]. Filtration of drinking water, restricting open defecation, establishing sufficient toilets, and educating people about hygiene behaviors are the other measures that help reduce the disease load [119].

Surveillance Programs

Managing typhoid is sometimes exacerbated by a lack of comprehensive countrywide surveillance in afflicted nations [74]. The Integrated Disease Surveillance Program initiated in 2004 aims to create and enhance a decentralized laboratory-based, computerized disease surveillance system to monitor 22 illnesses (including enteric fever) to identify and respond appropriately [74].

The National Surveillance System for Enteric Fever in India is a recent study in India investigating the prevalence of enteric fever in children aged six months to 15 years. The study uses active case-seeking methods to discover patients who might otherwise go unnoticed by facility-based monitoring systems [120].

The Surveillance of Enteric Fever in Asia Project, initiated by the Sabin Vaccine Institute in the United States, is a hospital-based enteric fever monitoring network in Asian nations, including India, Bangladesh, Indonesia, Pakistan, and Nepal [121]. The retrospective investigation in India yielded information about the disease prevalence, presentation, consequences, and antibiotic resistance trends [119]. This project helps with better preparation of future monitoring systems. Nations should routinely gather data on enteric fever to make evidence-based decisions about managing and preventing enteric fever [121].

The Severe Typhoid Fever Surveillance in Africa program stretched on the infrastructure established as part of the Typhoid Fever Surveillance in Africa Program study to portray the severity and long-term effects of typhoid fever and invasive nontyphoidal *Salmonella* disease across Africa. Between 2010 and 2014, multiple Sub-Saharan African countries gathered blood culture data and generated typhoid incidence rates from the site [122].

The Strategic Typhoid Alliance Across Africa and Asia consortium conducted a prospective multicomponent epidemiological study in Bangladesh, Nepal, and Malawi in three highly populated urban areas. From June 2016, passive surveillance continued at referral hospitals and primary healthcare facilities. Patients who presented with a fever of two days or longer or a recorded temperature of 38°C were enrolled, and blood cultures were collected. Age-stratified sero surveys were conducted at each research location to estimate the seroincidence of typhoid infection. The resultant seroincidence rates could evaluate and establish upper boundaries for the incidence rates determined from blood culture monitoring. Host responses serve as a proxy for infection incidence, and serosurveys can also detect the probable chronic carriers of *S.* Typhi. To discover stool shedding, people with a robust anti-Vi antibody will do a stool culture [123].

Some of the obstacles that surveillance systems encounter include inaccuracies in reporting fever, errors in diagnostic testing, the prolonged time required to report culture findings, shortage of diagnostic facilities in rural and isolated regions, and insufficiently trained laboratory staff [74].

Identification of various epidemiological features is critical in any surveillance system. Failure to examine these may result in a misunderstanding of disease burden, age distribution, cost of illness, transmission, and antibiotic susceptibility patterns [124].

Typhoid Vaccines

Vaccination is the primary strategy to lessen antibiotic dependency while also protecting against future antimicrobial resistance [125]. The early 1970s chloramphenicol-resistant typhoid outbreak in Mexico prompted a significant focus on vaccine research. It led to the creation of the live oral vaccine, Ty21a, and the non-denatured Vi polysaccharide vaccine [125].

Currently, three kinds of typhoid vaccinations are available for use: live oral Ty21a vaccine, parenteral Vi polysaccharide vaccine, and parenteral typhoid conjugate vaccine (TCV) [126].

Ty21a vaccine: Ty21a is a live vaccination that contains a mutant strain of *S.* Typhi (Ty21a) and is administered orally in three or four doses over five days. There is no approval for use in children under six [127]. The 2018 Cochrane report stated that one year after the Ty21a vaccination, effectiveness was 45%, and 59% after two years [128].

Vi polysaccharide vaccine: The Vi polysaccharide vaccine, developed in the 1980s, is a single-dose injectable vaccination containing purified Vi antigen. There is no approval for children under two, and because the immunological response is generally short-lived, we need to repeat the doses every two to three years [129]. A randomized, double-blind trial involving over 11,000 children in South Africa found 64% effectiveness after 21 months of surveillance [130]. In a study of 131,271 children and adults in China, an efficacy of 69 % was recorded [131]. According to an Indian study, mass vaccination with the Vi vaccine protects unvaccinated community members, with 44% efficacy recorded among non-vaccinated persons residing in vaccination regions. Children aged two to five years old had 80% protection [132]. The advantages of the Vi vaccine include a low side effect profile and convenience of administration (only one injection) [126].

However, following an initial peak post-vaccine, Vi antibody levels gradually declined; one study found that nearly two-thirds of patients had antibody titer below their predicted protective cut-off 27 months after vaccination [133]. Various animal investigations in the 1990s revealed that conjugating the Vi polysaccharide to a protein carrier boosted its immunogenicity [134].

TCVs: The WHO recommends TCVs for use in endemic regions, and they are the preferable vaccination in children as young as six months old [135]. Some examples of TCVs are Vi-rEPA TCV (Vi conjugated to recombinant exoprotein of *Pseudomonas aeruginosa*), Vi-Tetanus Toxoid TCV (Vi conjugated to tetanus toxoid), PedaTyph (Vi conjugated to tetanus toxoid), and Typbar TCV (Vi conjugated to tetanus toxoid) [126].

Vi-rEPA by the National Institutes of Health in the United States went through clinical studies in both the United States and Vietnam. However, they never scaled up the manufacturing to an industrial level [136]. In India, the PedaTyph vaccine and Typbar TCV got the license for use [117].

In a study involving 1,765 Indian children over six months, PedaTyph proved 100% efficient in preventing typhoid. Over 12 months, the vaccination group did not report any typhoid fever cases compared to a typhoid rate of 1.27% in the control group. At 12 months after vaccination, 83% of vaccinated children had significant antibody responses (a fourfold increase from baseline) [137].

Shakya et al. stated in 2019 that Typbar TCV revealed protective vaccination efficacy of 81.6% during a 15-month follow-up period in an interim analysis of a study including over 20,000 Nepali children [138].

There are numerous additional TCVs in various stages of development and those that are previously licensed. Vi-CRM197 from GlaxoSmithKline, a Vi-TT (tetanus toxoid) vaccine from Zydus Cadila, and Vi-DT (diphtheria toxoid) from the International Vaccine Institute are among them [139].

University of Maryland's Center for Vaccine Development generated live, attenuated oral vaccine strains, namely CVD 908, CVD 909, CVD 910, and CVD 915 [140]. The *S.* Typhi Ty2 strain served as the basis for all four strains [140]. CVD 909 was designed to produce Vi polysaccharides and stimulate Vi antibody responses and humoral and cell-mediated immune responses [141]. CVD 909 is an oral primer prior to parenteral Vi polysaccharide vaccine administration. In participants primed with CVD 909, Vi-specific IgA B memory cells were enhanced, suggesting that oral vaccinations might improve immunological memory [141].

While breakthroughs in water and sanitation have abolished endemic typhoid in many areas, numerous countries are still decades away from having enough water and sanitation to avoid typhoid fever. In such cases, antibiotics and vaccines are critical tools in the fight against typhoid fever.

## Conclusions

Enteric fever is still a significant cause of morbidity and mortality in developing countries. The diagnostic dilemma in most cases is mainly due to the diverse clinical presentation and lack of accurate diagnostic methods. The organism primarily affects the gastrointestinal tract and reticuloendothelial system. Literature has described multiple mechanisms responsible for the organism's Vi, like *Salmonella* pathogenicity islands, type III secretion mechanism, PAMPs, and secretion of AB toxin.

We have identified various genes responsible for the resistance of drugs like chloramphenicol, ampicillin, and trimethoprim-sulfamethoxazole. However, the increasing resistance to newer antibiotics is alarming and makes the management of enteric fever more complicated. The choice of antibiotic for enteric fever should be guided by local resistance patterns and susceptibility testing. It's crucial to consult with a healthcare professional or infectious disease specialist who is familiar with the current antibiotic resistance patterns in a specific region. However, combining fluoroquinolone with azithromycin or third-generation cephalosporin with azithromycin has become a recommended approach in some regions. Further research is still needed to comprehend drug resistance and develop new antimicrobials. The early diagnosis of enteric fever helps prompt treatment and prevention of chronic carrier state. New diagnostic methods like Typhidot, Tubex TF, TPTest, spectrometry, and PCR are up-and-coming with increased sensitivity. The benefit of these tests in resource-limited countries is debatable due to their limited accessibility and cost-effectiveness. Preventive strategies include implementing public health measures and immunizing the at-risk population, and proper surveillance plays a vital role in the long-term management of the disease. We recommend further surveillance studies in the endemic areas, which help identify the development of resistant strains and the effectiveness of the management policies.

## References

[1] Neupane DP, Dulal HP, Song J (2021). Enteric fever diagnosis: current challenges and future directions. Pathogens.

[2] (2019). The global burden of typhoid and paratyphoid fevers: a systematic analysis for the Global Burden of Disease Study 2017. Lancet Infect Dis.

[3] Gibani MM, Britto C, Pollard AJ (2018). Typhoid and paratyphoid fever: a call to action. Curr Opin Infect Dis.

[4] Frempong SN, King N, Sagoo GS (2022). The cost-effectiveness of using rapid diagnostic tests for the diagnosis of typhoid fever in patients with suspected typhoid fever: a systematic review. Expert Rev Pharmacoecon Outcomes Res.

[5] Azmatullah A, Qamar FN, Thaver D, Zaidi AK, Bhutta ZA (2015). Systematic review of the global epidemiology, clinical and laboratory profile of enteric fever. J Glob Health.

[6] Marchello CS, Birkhold M, Crump JA (2020). Complications and mortality of typhoid fever: a global systematic review and meta-analysis. J Infect.

[7] Cruz Espinoza LM, McCreedy E, Holm M (2019). Occurrence of typhoid fever complications and their relation to duration of illness preceding hospitalization: a systematic literature review and meta-analysis. Clin Infect Dis.

[8] (2003). Background document: the diagnosis, treatment and prevention of typhoid fever. https://www.glowm.com/pdf/WHO-diagnosis%20treatment%20prevention%20of%20typhoid%20fever-2003-CustomLicense.pdf.

[9] Cumming O, Elliott M, Overbo A, Bartram J (2014). Does global progress on sanitation really lag behind water? An analysis of global progress on community- and household-level access to safe water and sanitation. PLoS One.

[10] (2008). Typhoid vaccines: WHO position paper. Wkly Epidemiol Rec.

[11] Browne AJ, Kashef Hamadani BH, Kumaran EA (2020). Drug-resistant enteric fever worldwide, 1990 to 2018: a systematic review and meta-analysis. BMC Med.

[12] Dougan G, Baker S (2014). Salmonella enterica serovar Typhi and the pathogenesis of typhoid fever. Annu Rev Microbiol.

[13] Bueno SM, Santiviago CA, Murillo AA (2004). Precise excision of the large pathogenicity island, SPI7, in Salmonella enterica serovar Typhi. J Bacteriol.

[14] Dobinson HC, Gibani MM, Jones C (2017). Evaluation of the clinical and microbiological response to Salmonella Paratyphi a infection in the first paratyphoid human challenge model. Clin Infect Dis.

[15] Al-Sanouri TM, Paglietti B, Haddadin A, Murgia M, Bacciu D, Youssef M, Rubino S (2008). Emergence of plasmid-mediated multidrug resistance in epidemic and non-epidemic strains of Salmonella enterica serotype Typhi from Jordan. J Infect Dev Ctries.

[16] Kothari A, Pruthi A, Chugh TD (2008). The burden of enteric fever. J Infect Dev Ctries.

[17] Srikantiah P, Girgis FY, Luby SP (2006). Population-based surveillance of typhoid fever in Egypt. Am J Trop Med Hyg.

[18] Glynn JR, Hornick RB, Levine MM, Bradley DJ (1995). Infecting dose and severity of typhoid: analysis of volunteer data and examination of the influence of the definition of illness used. Epidemiol Infect.

[19] Coburn B, Grassl GA, Finlay BB (2007). Salmonella, the host and disease: a brief review. Immunol Cell Biol.

[20] de Jong HK, Parry CM, van der Poll T, Wiersinga WJ (2012). Host-pathogen interaction in invasive Salmonellosis. PLoS Pathog.

[21] Berrocal L, Fuentes JA, Trombert AN, Jofré MR, Villagra NA, Valenzuela LM, Mora GC (2015). stg fimbrial operon from S. Typhi STH2370 contributes to association and cell disruption of epithelial and macrophage-like cells. Biol Res.

[22] Harris JC, Dupont HL, Hornick RB (1972). Fecal leukocytes in diarrheal illness. Ann Intern Med.

[23] Vazquez-Torres A, Jones-Carson J, Bäumler AJ (1999). Extraintestinal dissemination of Salmonella by CD18-expressing phagocytes. Nature.

[24] Lemire S, Figueroa-Bossi N, Bossi L (2008). A singular case of prophage complementation in mutational activation of recET orthologs in Salmonella enterica serovar Typhimurium. J Bacteriol.

[25] Su LH, Wu TL, Chia JH, Chu C, Kuo AJ, Chiu CH (2005). Increasing ceftriaxone resistance in Salmonella isolates from a university hospital in Taiwan. J Antimicrob Chemother.

[26] Hart PJ, O'Shaughnessy CM, Siggins MK (2016). Differential killing of Salmonella enterica serovar Typhi by antibodies targeting vi and lipopolysaccharide o:9 antigen. PLoS One.

[27] Pham Thanh D, Thompson CN, Rabaa MA (2016). The molecular and spatial epidemiology of typhoid fever in rural Cambodia. PLoS Negl Trop Dis.

[28] Coburn B, Sekirov I, Finlay BB (2007). Type III secretion systems and disease. Clin Microbiol Rev.

[29] Bishop A, House D, Perkins T, Baker S, Kingsley RA, Dougan G (2008). Interaction of Salmonella enterica serovar Typhi with cultured epithelial cells: roles of surface structures in adhesion and invasion. Microbiology (Reading).

[30] Mohanty S, Gaind R, Sehgal R, Chellani H, Deb M (2009). Neonatal sepsis due to Salmonella Typhi and Paratyphi A. J Infect Dev Ctries.

[31] Ramos-Morales F (2012). Impact of Salmonella enterica type III secretion system effectors on the eukaryotic host cell. ISRN Cell Biol.

[32] Zaki SA, Shanbag P (2010). Clinical manifestations of dengue and leptospirosis in children in Mumbai: an observational study. Infection.

[33] Gaind R, Paglietti B, Murgia M (2006). Molecular characterization of ciprofloxacin-resistant Salmonella enterica serovar Typhi and Paratyphi A causing enteric fever in India. J Antimicrob Chemother.

[34] Perez-Lopez A, Behnsen J, Nuccio SP, Raffatellu M (2016). Mucosal immunity to pathogenic intestinal bacteria. Nat Rev Immunol.

[35] Lee S, Yang YA, Milano SK (2020). Salmonella typhoid toxin pltb subunit and its non-typhoidal Salmonella ortholog confer differential host adaptation and virulence. Cell Host Microbe.

[36] Zeng H, Carlson AQ, Guo Y (2003). Flagellin is the major proinflammatory determinant of enteropathogenic Salmonella. J Immunol.

[37] Winter SE, Raffatellu M, Wilson RP, Rüssmann H, Bäumler AJ (2008). The Salmonella enterica serotype Typhi regulator TviA reduces interleukin-8 production in intestinal epithelial cells by repressing flagellin secretion. Cell Microbiol.

[38] Song J, Gao X, Galán JE (2013). Structure and function of the Salmonella Typhi chimaeric A(2)B(5) typhoid toxin. Nature.

[39] Chang SJ, Song J, Galán JE (2016). Receptor-mediated sorting of typhoid toxin during its export from Salmonella Typhi-infected cells. Cell Host Microbe.

[40] Hiyoshi H, Wangdi T, Lock G, Saechao C, Raffatellu M, Cobb BA, Bäumler AJ (2018). Mechanisms to evade the phagocyte respiratory burst arose by convergent evolution in typhoidal Salmonella serovars. Cell Rep.

[41] Raffatellu M, Chessa D, Wilson RP, Dusold R, Rubino S, Bäumler AJ (2005). The Vi capsular antigen of Salmonella enterica serotype Typhi reduces Toll-like receptor-dependent interleukin-8 expression in the intestinal mucosa. Infect Immun.

[42] McCormick BA, Miller SI, Carnes D, Madara JL (1995). Transepithelial signaling to neutrophils by salmonellae: a novel virulence mechanism for gastroenteritis. Infect Immun.

[43] Sulaiman K, Sarwari AR (2007). Culture-confirmed typhoid fever and pregnancy. Int J Infect Dis.

[44] Lewis MD, Serichantalergs O, Pitarangsi C (2005). Typhoid fever: a massive, single-point source, multidrug-resistant outbreak in Nepal. Clin Infect Dis.

[45] Ochiai RL, Acosta CJ, Danovaro-Holliday MC (2008). A study of typhoid fever in five Asian countries: disease burden and implications for controls. Bull World Health Organ.

[46] Zaki SA, Karande S (2011). Multidrug-resistant typhoid fever: a review. J Infect Dev Ctries.

[47] Yoo S, Pai H, Byeon JH, Kang YH, Kim S, Lee BK (2004). Epidemiology of Salmonella enterica serotype typhi infections in Korea for recent 9 years: trends of antimicrobial resistance. J Korean Med Sci.

[48] Bhutta ZA (2006). Current concepts in the diagnosis and treatment of typhoid fever. BMJ.

[49] Chen HM, Wang Y, Su LH, Chiu CH (2013). Nontyphoid salmonella infection: microbiology, clinical features, and antimicrobial therapy. Pediatr Neonatol.

[50] (2003). Background document: the diagnosis, treatment and prevention of typhoid fever. https://www.glowm.com/pdf/WHO-diagnosis%20treatment%20prevention%20of%20typhoid%20fever-2003-CustomLicense.pdf.

[51] Capoor MR, Rawat D, Nair D, Hasan AS, Deb M, Aggarwal P, Pillai P (2007). In vitro activity of azithromycin, newer quinolones and cephalosporins in ciprofloxacin-resistant Salmonella causing enteric fever. J Med Microbiol.

[52] Turner AK, Nair S, Wain J (2006). The acquisition of full fluoroquinolone resistance in Salmonella Typhi by accumulation of point mutations in the topoisomerase targets. J Antimicrob Chemother.

[53] Wong VK, Baker S, Pickard DJ (2015). Phylogeographical analysis of the dominant multidrug-resistant H58 clade of Salmonella Typhi identifies inter- and intracontinental transmission events. Nat Genet.

[54] Wong VK, Baker S, Pickard D, Page AJ, Feasey NA, Dougan G, Holt KE (2016). The emergence and intercontinental spread of a multidrug-resistant clade of typhoid agent Salmonella enterica serovar Typhi. Lancet.

[55] Gupta V, Kaur J, Kaistha N (2009). Re-emerging chloramphenicol sensitivity and emerging low level ciprofloxacin resistance among Salmonella enterica serotype typhi isolates in North India. Trop Doct.

[56] Savoldi A, Carrara E, Gladstone BP, Azzini AM, Göpel S, Tacconelli E (2019). Gross national income and antibiotic resistance in invasive isolates: analysis of the top-ranked antibiotic-resistant bacteria on the 2017 WHO priority list. J Antimicrob Chemother.

[57] Yan M, Li X, Liao Q, Li F, Zhang J, Kan B (2016). The emergence and outbreak of multidrug-resistant typhoid fever in China. Emerg Microbes Infect.

[58] Arjyal A, Basnyat B, Nhan HT (2016). Gatifloxacin versus ceftriaxone for uncomplicated enteric fever in Nepal: an open-label, two-centre, randomised controlled trial. Lancet Infect Dis.

[59] Kalra SP, Naithani N, Mehta SR, Swamy AJ (2003). Current trends in the management of typhoid fever. Med J Armed Forces India.

[60] Klemm EJ, Shakoor S, Page AJ (2018). Emergence of an extensively drug-resistant Salmonella enterica serovar Typhi clone harboring a promiscuous plasmid encoding resistance to fluoroquinolones and third-generation cephalosporins. mBio.

[61] Kaurthe J (2013). Increasing antimicrobial resistance and narrowing therapeutics in typhoidal salmonellae. J Clin Diagn Res.

[62] Pfeifer Y, Matten J, Rabsch W (2009). Salmonella enterica serovar Typhi with CTX-M beta-lactamase, Germany. Emerg Infect Dis.

[63] Gokul BN, Menezes GA, Harish BN (2010). ACC-1 beta-lactamase-producing Salmonella enterica Serovar Typhi, India. Emerg Infect Dis.

[64] Misra R, Prasad KN (2016). Antimicrobial susceptibility to azithromycin among Salmonella enterica Typhi and Paratyphi A isolates from India. J Med Microbiol.

[65] Sakamoto N, Nakamura-Uchiyama F, Kobayashi K (2013). Severe murine typhus with shock and acute respiratory failure in a Japanese traveler after returning from Thailand. J Travel Med.

[66] Hassing RJ, Goessens WH, van Pelt W (2014). Salmonella subtypes with increased MICs for azithromycin in travelers returned to The Netherlands. Emerg Infect Dis.

[67] Capoor MR, Nair D, Posti J, Singhal S, Deb M, Aggarwal P, Pillai P (2009). Minimum inhibitory concentration of carbapenems and tigecycline against Salmonella spp. J Med Microbiol.

[68] Tunger O, Karakaya Y, Cetin CB, Dinc G, Borand H (2009). Rational antibiotic use. J Infect Dev Ctries.

[69] Sheikh A, Bhuiyan MS, Khanam F (2009). Salmonella enterica serovar Typhi-specific immunoglobulin A antibody responses in plasma and antibody in lymphocyte supernatant specimens in Bangladeshi patients with suspected typhoid fever. Clin Vaccine Immunol.

[70] Kariuki S, Revathi G, Kiiru J (2010). Typhoid in Kenya is associated with a dominant multidrug-resistant Salmonella enterica serovar Typhi haplotype that is also widespread in Southeast Asia. J Clin Microbiol.

[71] Meltzer E, Stienlauf S, Leshem E, Sidi Y, Schwartz E (2014). A large outbreak of Salmonella Paratyphi A infection among israeli travelers to Nepal. Clin Infect Dis.

[72] Chan BK, Abedon ST (2012). Phage therapy pharmacology phage cocktails. Adv Appl Microbiol.

[73] Parracho HM, Burrowes BH, Enright MC, McConville ML, Harper DR (2012). The role of regulated clinical trials in the development of bacteriophage therapeutics. J Mol Genet Med.

[74] Mukhopadhyay B, Sur D, Gupta SS, Ganguly NK (2019). Typhoid fever: control &amp; challenges in India. Indian J Med Res.

[75] Waddington CS, Darton TC, Pollard AJ (2014). The challenge of enteric fever. J Infect.

[76] Kumar P, Kumar R (2017). Enteric fever. Indian J Pediatr.

[77] Sümer A, Kemik O, Dülger AC (2010). Outcome of surgical treatment of intestinal perforation in typhoid fever. World J Gastroenterol.

[78] Atamanalp SS, Aydinli B, Ozturk G, Oren D, Basoglu M, Yildirgan MI (2007). Typhoid intestinal perforations: twenty-six year experience. World J Surg.

[79] Crump JA, Luby SP, Mintz ED (2004). The global burden of typhoid fever. Bull World Health Organ.

[80] Baker S, Favorov M, Dougan G (2010). Searching for the elusive typhoid diagnostic. BMC Infect Dis.

[81] Sejvar J, Lutterloh E, Naiene J (2012). Neurologic manifestations associated with an outbreak of typhoid fever, Malawi--Mozambique, 2009: an epidemiologic investigation. PLoS One.

[82] Crump JA, Sjölund-Karlsson M, Gordon MA, Parry CM (2015). Epidemiology, clinical presentation, laboratory diagnosis, antimicrobial resistance, and antimicrobial management of invasive Salmonella infections. Clin Microbiol Rev.

[83] Crump JA (2012). Typhoid fever and the challenge of nonmalaria febrile illness in sub-saharan Africa. Clin Infect Dis.

[84] Pickard D, Wain J, Baker S (2003). Composition, acquisition, and distribution of the Vi exopolysaccharide-encoding Salmonella enterica pathogenicity island SPI-7. J Bacteriol.

[85] Lanata CF, Levine MM, Ristori C (1983). Vi serology in detection of chronic Salmonella typhi carriers in an endemic area. Lancet.

[86] Pickard D, Li J, Roberts M (1994). Characterization of defined ompR mutants of Salmonella typhi: ompR is involved in the regulation of Vi polysaccharide expression. Infect Immun.

[87] Brewer SM, Twittenhoff C, Kortmann J (2021). A Salmonella Typhi RNA thermosensor regulates virulence factors and innate immune evasion in response to host temperature. PLoS Pathog.

[88] Baker S, Hardy J, Sanderson KE (2007). A novel linear plasmid mediates flagellar variation in Salmonella Typhi. PLoS Pathog.

[89] Levine MM, Grados O, Gilman RH, Woodward WE, Solis-Plaza R, Waldman W (1978). Diagnostic value of the Widal test in areas endemic for typhoid fever. Am J Trop Med Hyg.

[90] Olopoenia LA, King AL (2000). Widal agglutination test - 100 years later: still plagued by controversy. Postgrad Med J.

[91] House D, Chinh NT, Diep TS (2005). Use of paired serum samples for serodiagnosis of typhoid fever. J Clin Microbiol.

[92] Parry CM, Hoa NT, Diep TS (1999). Value of a single-tube widal test in diagnosis of typhoid fever in Vietnam. J Clin Microbiol.

[93] Kulkarni ML, Rego SJ (1994). Value of single Widal test in the diagnosis of typhoid fever. Indian Pediatr.

[94] Olsen SJ, Pruckler J, Bibb W (2004). Evaluation of rapid diagnostic tests for typhoid fever. J Clin Microbiol.

[95] Gopalakrishnan V, Sekhar WY, Soo EH, Vinsent RA, Devi S (2002). Typhoid fever in Kuala Lumpur and a comparative evaluation of two commercial diagnostic kits for the detection of antibodies to Salmonella Typhi. Singapore Med J.

[96] Kawano RL, Leano SA, Agdamag DM (2007). Comparison of serological test kits for diagnosis of typhoid fever in the Philippines. J Clin Microbiol.

[97] Anusha R, Ganesh R, Lalitha J (2011). Comparison of a rapid commercial test, Enterocheck WB(®), with automated blood culture for diagnosis of typhoid fever. Ann Trop Paediatr.

[98] Helman SK, Mummah RO, Gostic KM, Buhnerkempe MG, Prager KC, Lloyd-Smith JO (2020). Estimating prevalence and test accuracy in disease ecology: how Bayesian latent class analysis can boost or bias imperfect test results. Ecol Evol.

[99] Islam K, Sayeed MA, Hossen E (2016). Comparison of the performance of the TPTest, Tubex, Typhidot and Widal immunodiagnostic assays and blood cultures in detecting patients with typhoid fever in Bangladesh, including using a Bayesian latent class modeling approach. PLoS Negl Trop Dis.

[100] Arora P, Thorlund K, Brenner DR, Andrews JR (2019). Comparative accuracy of typhoid diagnostic tools: a Bayesian latent-class network analysis. PLoS Negl Trop Dis.

[101] Moore CE, Pan-Ngum W, Wijedoru LP (2014). Evaluation of the diagnostic accuracy of a typhoid IgM flow assay for the diagnosis of typhoid fever in Cambodian children using a Bayesian latent class model assuming an imperfect gold standard. Am J Trop Med Hyg.

[102] Storey HL, Huang Y, Crudder C, Golden A, de los Santos T, Hawkins K (2015). A meta-analysis of typhoid diagnostic accuracy studies: a recommendation to adopt a standardized composite reference. PLoS One.

[103] Näsström E, Vu Thieu NT, Dongol S (2014). Salmonella Typhi and Salmonella Paratyphi A elaborate distinct systemic metabolite signatures during enteric fever. Elife.

[104] Darton TC, Blohmke CJ, Giannoulatou E (2015). Rapidly escalating hepcidin and associated serum iron starvation are features of the acute response to typhoid infection in humans. PLoS Negl Trop Dis.

[105] Blohmke CJ, Darton TC, Jones C (2016). Interferon-driven alterations of the host's amino acid metabolism in the pathogenesis of typhoid fever. J Exp Med.

[106] Khanam F, Sheikh A, Sayeed MA (2013). Evaluation of a typhoid/paratyphoid diagnostic assay (TPTest) detecting anti-Salmonella IgA in secretions of peripheral blood lymphocytes in patients in Dhaka, Bangladesh. PLoS Negl Trop Dis.

[107] Liang L, Juarez S, Nga TV (2013). Immune profiling with a Salmonella Typhi antigen microarray identifies new diagnostic biomarkers of human typhoid. Sci Rep.

[108] Charles RC, Liang L, Khanam F (2014). Immunoproteomic analysis of antibody in lymphocyte supernatant in patients with typhoid fever in Bangladesh. Clin Vaccine Immunol.

[109] Darton TC, Baker S, Randall A (2017). Identification of novel serodiagnostic signatures of typhoid fever using a salmonella proteome array. Front Microbiol.

[110] Charles RC, Sheikh A, Krastins B (2010). Characterization of anti-Salmonella enterica serotype Typhi antibody responses in bacteremic Bangladeshi patients by an immunoaffinity proteomics-based technology. Clin Vaccine Immunol.

[111] Blohmke CJ, Muller J, Gibani MM (2019). Diagnostic host gene signature for distinguishing enteric fever from other febrile diseases. EMBO Mol Med.

[112] Song JH, Cho H, Park MY, Na DS, Moon HB, Pai CH (1993). Detection of Salmonella typhi in the blood of patients with typhoid fever by polymerase chain reaction. J Clin Microbiol.

[113] Hatta M, Smits HL (2007). Detection of Salmonella typhi by nested polymerase chain reaction in blood, urine, and stool samples. Am J Trop Med Hyg.

[114] Sánchez-Jiménez MM, Cardona-Castro N (2004). Validation of a PCR for diagnosis of typhoid fever and salmonellosis by amplification of the hilA gene in clinical samples from Colombian patients. J Med Microbiol.

[115] Nandagopal B, Sankar S, Lingesan K, Appu KC, Padmini B, Sridharan G, Gopinath AK (2010). Prevalence of Salmonella typhi among patients with febrile illness in rural and peri-urban populations of Vellore district, as determined by nested PCR targeting the flagellin gene. Mol Diagn Ther.

[116] Chaudhry R, Chandel DS, Verma N, Singh N, Singh P, Dey AB (2010). Rapid diagnosis of typhoid fever by an in-house flagellin PCR. J Med Microbiol.

[117] Wain J, Hendriksen RS, Mikoleit ML, Keddy KH, Ochiai RL (2015). Typhoid fever. Lancet.

[118] (2019). Progress on drinking water, sanitation and hygiene 2000-2017: special focus on inequalities. https://www.unicef.org/reports/progress-on-drinking-water-sanitation-and-hygiene-2019..

[119] Bennett SD, Lowther SA, Chingoli F (2018). Assessment of water, sanitation and hygiene interventions in response to an outbreak of typhoid fever in Neno District, Malawi. PLoS One.

[120] John J, Bavdekar A, Rongsen-Chandola T, Dutta S, Kang G (2018). Estimating the incidence of enteric fever in children in India: a multi-site, active fever surveillance of pediatric cohorts. BMC Public Health.

[121] Sur D, Barkume C, Mukhopadhyay B, Date K, Ganguly NK, Garrett D (2018). A retrospective review of hospital-based data on enteric fever in india, 2014-2015. J Infect Dis.

[122] Carey ME, MacWright WR, Im J (2020). The Surveillance for Enteric Fever in Asia Project (SEAP), Severe Typhoid Fever Surveillance in Africa (SETA), Surveillance of Enteric Fever in India (SEFI), and Strategic Typhoid Alliance Across Africa and Asia (STRATAA) Population-based Enteric Fever Studies: a review of methodological similarities and differences. Clin Infect Dis.

[123] Darton TC, Meiring JE, Tonks S (2017). The STRATAA study protocol: a programme to assess the burden of enteric fever in Bangladesh, Malawi and Nepal using prospective population census, passive surveillance, serological studies and healthcare utilisation surveys. BMJ Open.

[124] Saha S, Islam M, Saha S (2018). Designing comprehensive public health surveillance for enteric fever in endemic countries: importance of including different healthcare facilities. J Infect Dis.

[125] Pang T, Levine MM, Ivanoff B, Wain J, Finlay BB (1998). Typhoid fever--important issues still remain. Trends Microbiol.

[126] O'Reilly PJ, Pant D, Shakya M, Basnyat B, Pollard AJ (2020). Progress in the overall understanding of typhoid fever: implications for vaccine development. Expert Rev Vaccines.

[127] Amicizia D, Arata L, Zangrillo F, Panatto D, Gasparini R (2017). Overview of the impact of typhoid and paratyphoid fever. Utility of Ty21a vaccine (Vivotif®). J Prev Med Hyg.

[128] Milligan R, Paul M, Richardson M, Neuberger A (2018). Vaccines for preventing typhoid fever. Cochrane Database Syst Rev.

[129] Date KA, Bentsi-Enchill A, Marks F, Fox K (2015). Typhoid fever vaccination strategies. Vaccine.

[130] Klugman KP, Koornhof HJ, Schneerson R (1987). Protective activity of Vi capsular polysaccharide vaccine against typhoid fever. Lancet.

[131] Yang HH, Wu CG, Xie GZ (2001). Efficacy trial of Vi polysaccharide vaccine against typhoid fever in south-western China. Bull World Health Organ.

[132] Sur D, Ochiai RL, Bhattacharya SK (2009). A cluster-randomized effectiveness trial of Vi typhoid vaccine in India. N Engl J Med.

[133] Keitel WA, Bond NL, Zahradnik JM, Cramton TA, Robbins JB (1994). Clinical and serological responses following primary and booster immunization with Salmonella typhi Vi capsular polysaccharide vaccines. Vaccine.

[134] Szu SC, Stone AL, Robbins JD, Schneerson R, Robbins JB (1987). Vi capsular polysaccharide-protein conjugates for prevention of typhoid fever. Preparation, characterization, and immunogenicity in laboratory animals. J Exp Med.

[135] (2019). Typhoid vaccines: WHO position paper, March 2018 - recommendations. Vaccine.

[136] Martin LB (2012). Vaccines for typhoid fever and other salmonelloses. Curr Opin Infect Dis.

[137] Mitra M, Shah N, Ghosh A, Chatterjee S, Kaur I, Bhattacharya N, Basu S (2016). Efficacy and safety of vi-tetanus toxoid conjugated typhoid vaccine (PedaTyph™) in Indian children: school based cluster randomized study. Hum Vaccin Immunother.

[138] Shakya M, Colin-Jones R, Theiss-Nyland K (2019). Phase 3 efficacy analysis of a typhoid conjugate vaccine trial in Nepal. N Engl J Med.

[139] Sahastrabuddhe S, Saluja T (2019). Overview of the typhoid conjugate vaccine pipeline: current status and future plans. Clin Infect Dis.

[140] Salerno-Gonçalves R, Galen JE, Levine MM, Fasano A, Sztein MB (2018). Manipulation of Salmonella Typhi gene expression impacts innate cell responses in the human intestinal mucosa. Front Immunol.

[141] Wahid R, Pasetti MF, Maciel M Jr, Simon JK, Tacket CO, Levine MM, Sztein MB (2011). Oral priming with Salmonella Typhi vaccine strain CVD 909 followed by parenteral boost with the S. Typhi Vi capsular polysaccharide vaccine induces CD27+IgD-S. Typhi-specific IgA and IgG B memory cells in humans. Clin Immunol.

